# Practical considerations for a TB controlled human infection model (TB-CHIM); the case for TB-CHIM in Africa, a systematic review of the literature and report of 2 workshop discussions in UK and Malawi

**DOI:** 10.12688/wellcomeopenres.18767.1

**Published:** 2023-02-10

**Authors:** Stephen B. Gordon, Simon Sichone, Anthony E. Chirwa, Phoebe Hazenberg, Zacharia Kafuko, Daniela M. Ferreira, Joanne Flynn, Sarah Fortune, Shobana Balasingam, Giancarlo A. Biagini, Helen McShane, Henry Mwandumba, Kondwani Jambo, Keertan Dedha, Nimisha Raj Sharma, Brian D. Robertson, Naomi Walker, Ben Morton, TB Controlled Human Infection Model Development Group

**Affiliations:** 1 Malawi Liverpool Wellcome Trust Clinical Research Programme, Blantyre, Malawi; 2 Liverpool School of Tropical Medicine, Liverpool, L3 5QA, UK; 3 1Day Africa, 1Day Sooner, Lusaka Province, Zambia; 4 Oxford Vaccine Group, University of Oxford, Oxford, UK; 5 Centre for Vaccine Research, University of Pittsburgh, Pittsburgh, Pennsylvania, USA; 6 Harvard T.H. Chan School of Public Health, Harvard University, Boston, Massachusetts, USA; 7 Wellcome Trust, London, UK; 8 The Jenner Institute, University of Oxford, Oxford, UK; 9 University of Cape Town, Cape Town, South Africa; 10 Imperial College London, London, UK

**Keywords:** Human infection studies, tuberculosis, TB, BCG, bacille Calmette Guerin, vaccine, immunology, genetically modified organism

## Abstract

**Background:** Tuberculosis (TB) remains a major challenge in many domains including diagnosis, pathogenesis, prevention, treatment, drug resistance and long-term protection of the public health by vaccination. A controlled human infection model (CHIM) could potentially facilitate breakthroughs in each of these domains but has so far been considered impossible owing to technical and safety concerns.

**Methods:** A systematic review of mycobacterial human challenge studies was carried out to evaluate progress to date, best possible ways forward and challenges to be overcome. We searched MEDLINE (1946 to current) and CINAHL (1984 to current) databases; and Google Scholar to search citations in selected manuscripts. The final search was conducted 3 ^rd ^February 2022. Inclusion criteria: adults ≥18 years old; administration of live mycobacteria; and interventional trials or cohort studies with immune and/or microbiological endpoints. Exclusion criteria: animal studies; studies with no primary data; no administration of live mycobacteria; retrospective cohort studies; case-series; and case-reports. Relevant tools (Cochrane Collaboration for RCTs and Newcastle-Ottawa Scale for non-randomised studies) were used to assess risk of bias and present a narrative synthesis of our findings.

**Results:** The search identified 1,388 titles for review; of these 90 were reviewed for inclusion; and 27 were included. Of these, 15 were randomised controlled trials and 12 were prospective cohort studies. We focussed on administration route, challenge agent and dose administered for data extraction. Overall, BCG studies including fluorescent BCG show the most immediate utility, and genetically modified *Mycobacteria tuberculosis* is the most tantalising prospect of discovery breakthrough.

**Conclusions:** The TB-CHIM development group met in 2019 and 2022 to consider the results of the systematic review, to hear presentations from many of the senior authors whose work had been reviewed and to consider best ways forward. This paper reports both the systematic review and the deliberations.

**Registration:** PROSPERO ( CRD42022302785; 21 January 2022).

## Introduction

### Need for a tuberculosis (TB) vaccine and challenges in development

More than 1.5 million people died of tuberculosis (TB) in 2021, of whom 214,000 were individuals living with HIV ^1^. An estimated 9.9 million people worldwide became ill with TB, with the greatest burden of this disease in Africa and Southeast Asia, and with 47% of TB-affected households suffering catastrophic costs ^1^. The COVID pandemic has reversed years of progress in global targets to end TB, making the search for an effective vaccine even more urgent ^2, 3^.

The Bacille Calmette-Guérin (BCG) vaccine was the first, and is still a century later, the only vaccine approved for humans that protects against TB. BCG is an attenuated *Mycobacterium bovis* strain with considerable sequence homology with *M. tuberculosis.* BCG is used both as a vaccine and as immunotherapy. BCG vaccine efficacy is highly variable in adults (0–80%) being most effective at high latitudes and particularly poor in tropical and subtropical regions ^4, 5^.

There are several new TB vaccines in development as listed in Table 1, targeting initial infection, disease, or drug resistant infections ^6^. Vaccine trial design is very challenging because initial infection is common in early life. This sensitisation is often partially immunising but not always sterilising ^7^, leaving both reinfection or recrudescent disease a risk for some years after initial infection ^8^ and making vaccine trial clinical endpoints very difficult to define. In a recent vaccine trial, the M72/AS01E candidate vaccine demonstrated 49.7% efficacy against microbiologically proven pulmonary TB disease at 3 year follow up in a phase 2b clinical trial ^9^ of more than 3,500 BCG-vaccinated subjects recruited with evidence of prior TB sensitisation (positive interferon gamma release assay (IGRA)). Now, a very large study is required for full evaluation of the clinical impact for this vaccine and the cost has been estimated at GBP 400 million.

**Table 1.** TB Vaccine candidates. Sources: 1- WHO Global TB report 2020, J.Li TB vaccine development: from classic to clinical candidates, 2- European Journal of Clinical Microbiology & Infectious Diseases, 2020, 3- Clinical trials.gov. TBVI: TuBerculosis Vaccine Initiative; IDRI: Infectious Disease Research Institute; RIBSP: Research Institute for Biological Safety Problems; MoH: Ministry of Health; ID: intradermal; IM: intramuscular; GHIT: Global Health Innovative Technology Fund; IAVI: International AIDS Vaccine Initiative; SSI: Statens Serum Institut; GSK: GlaxoSmithKline; MRI: Medical Research Institute; SIIPL: Serum Institute of India Pvt. Ltd; VPM: Vakzine Projekt Management; ICMR: Indian Council of Medical Research; TB: *Mycobacterium tuberculosis*; BCG: bacille Calmette-Guerin; MDR: multi drug resistant; and HIV: human immunodeficiency virus.

Latest Phase	Vaccine candidate	Vaccine platform	Target population
**I**	**Ad5 Ag85A** McMaster, CanSino	Viral vector	• Booster vaccine for adults with latent TB ( NCT02337270)
**IIa**	**AEC/BC02** Anhui Zhifei Longcom	Mycobacterial – whole cell or extract	• Booster vaccine for adults with latent TB ( NCT05284812)
	**MTBVAC** Biofabri, TBVI University of Zaragoza	Mycobacterial – live	• BCG replacement vaccine for infants ( NCT03536117), with a Phase III planned ( NCT04975178) • Adults with latent TB ( NCT02933281)
	**ID93 + GLA-SE** IDRI, Wellcome Trust	Protein/adjuvant	• Booster vaccine for adults with latent TB ( NCT02465216) • BCG vaccinated healthcare workers ( NCT03806686)
	**TB/FLU-04L** RIBSP	Viral vector	• Booster vaccine for adults with latent TB ( NCT02501421)
	**GamTBvac** MoH, Russian Federation	Protein/adjuvant	• BCG vaccinated adults ( NCT03878004), Phase III planned ( NCT04975737)
**IIb**	**ChAdOx185A-MVA85A** (ID, IM, Aerosol) University of Oxford	Viral vector	• Booster vaccine for infants ( NCT00953927), children ( NCT00679159) adolescents ( NCT02178748) Booster vaccine for adults with HIV and latent TB ( NCT01151189)
	**H4:IC-31** (Aeras‑404) IAVI, Sanofi Pasteur and Intercell	Protein/adjuvant	• Booster vaccine for infants ( NCT01861730), children and adolescents with BCG ( NCT02075203)
	**DAR-901** Booster Dartmouth, GHIT	Mycobacterial – whole cell or extract	• Booster vaccine for adolescents ( NCT02712424) • Booster vaccine for adults with latent TB and HIV ( NCT00052195)
	**H56:IC31** SSI, Valneva, IAVI	Protein/adjuvant	• Booster vaccine for previously infected adults ( NCT03512249) and adults with latent TB ( NCT01865487)
	**M72/AS01E** GSK, Gates MRI	Protein/adjuvant	• Booster vaccine for adults with latent TB ( NCT01755598) and HIV ( NCT04556981)
	**BCG** revaccination Gates MRI	Mycobacterial – live	• Booster vaccine for adolescents ( NCT04152161)
	**RUTI** Archivel Farma, S.L	Mycobacterial – whole cell or extract	• Booster vaccine for MDR-TB ( NCT04919239) and with latent TB ( NCT01136161)
**III**	**VPM1002** SIIPL, VPM	Mycobacterial – live	• BCG replacement vaccine for infants ( NCT02391415), with a planned Phase III ( NCT04351685) • Phase II/III trial planned for prevention of recurrence in adults previously treated with pulmonary TB ( NCT03152903)
	**MIP/Immunovac** ICMR, Cadila Pharmaceuticals	Mycobacterial – whole cell or extract	• Therapeutic vaccine for adults with active TB ( NCT00265226)
	**Vaccae** Anhui Zhiefei, Longcom, Biopharmacuetical Co, Ltd	Mycobacterial – whole cell or extract	• Therapeutic vaccine for adults with active TB ( NCT01979900), with MDR-TB and HIV (NCT01977768), and prevention of disseminated TB in HIV adults ( NCT00052195)
	**SRL172** IAVI	Mycobacterial – whole cell or extract	• Booster vaccine for adults with latent TB and HIV ( NCT00052195)

Given the wide choice of potential vaccines, uncertainty about vaccine trial endpoints, and the huge cost of vaccine trials against disease, precise tools are required to select the most promising new vaccine candidates for clinical evaluation. As it is very difficult to detect viable organisms in subjects with TB, an alternative vaccine trial design might be to rely on immunological endpoints demonstrating infection—but these endpoints may be similar in patients experiencing sterilising immunity or even subclinical disease ^10^. Controlled human infection models (CHIM), also called Human Infection Studies (HIS) might offer a further alternative to down-select vaccine candidates prior to phase 2b/3 efficacy trials. In CHIM trials, vaccines are tested for protection against experimental infection managed in volunteer subjects. An optimised TB-CHIM would help with vaccine selection, and identification of immune correlates. It would have potential to predict the efficacy of new drug regimens, and even offer discovery opportunities regarding pathogenesis and correlates of immunity ^11^.

### Potential for a TB-CHIM

CHIM have been successfully employed for many decades to accelerate vaccine development in enteric infections, and to select appropriate clinical trials in vaccines against other infections ^11^. Very recently, CHIM studies of SARS-CoV-2 have contributed substantially to knowledge of pathogenesis, infectivity, diagnostic precision of lateral flow tests and post-exposure protection against disease ^12^. Notable examples of recent vaccines that are being rolled out after clinical development accelerated by CHIM are the RTS,S/AS01 E malaria vaccine ^13^ and the typhoid conjugate vaccine ^14^.

There are significant challenges, however, for a *Mycobacterium tuberculosis (M.tb)* or TB CHIM (TB-CHIM). First, and most importantly, is the current concern for safety using wild-type *M.tb* as the infection cannot be reliably eradicated from an infected subject; treatment is prolonged (6 months) and toxic with potential for serious adverse events; and there is also potential risk of immunopathology for which treatment options may be inadequate or complicated. There is a small risk of recurrent infection (~12% cases) after treatment, highest in the first year and subsequently receding ^8^. There are reasons for optimism, however, that a conditionally replicating wild-type mycobacterium, including genetically inserted suicide switches to ensure complete sterility post-infection could become available and transform the study of human TB infection ^15^. In the meantime, there are sufficient studies of BCG to show that this organism induces anti-mycobacterial immunity and could serve as a model of wild-type infection, useful for vaccine testing provided that the vaccine mechanism was not unique to *M.tb* ^16, 17^. Further, there are modified BCG models, including those with included fluorophores, that will allow diverse endpoint measurements to alleviate the current difficulty in precise microbiological detection in the site of infection and remote sites ^18, 19^. Opportunities and limitations of the BCG models tested are discussed later in this review.

### Need for a TB-CHIM in Africa

There is a reasonable and strong drive to deliver CHIMs in infection endemic settings so that vaccines, drugs and therapeutics are directly tailored to the populations that need them most ^20^. This drive is particularly marked in infections where prior exposure and the intensity of community infection define the immunological context in which novel vaccines would be used. In addition, there is political momentum to ensure that the delivery of these needed vaccines to low and middle-income countries (LMIC) is not hindered by economic or regulatory factors arising in the country of invention. Recently, there has been considerable progress in delivering CHIM studies in Africa—examples include both falciparum and vivax malaria, pneumococcus, *N.lactamica* and schistosomiasis.

Exposure to both *M.tb* and non-tuberculous mycobacteria (NTM) are very common in Africa, often occurring at a very young age. Recurrent TB exposure occurs throughout life owing to late diagnosis of infectious cases who circulate in the community. Exposures that increase susceptibility to mycobacterial infection are common. These include but are not limited to malnutrition, smoke exposure, HIV, and potentially, other infections. In Africa, a TB-CHIM could be transformative for testing of vaccines, early-stage drug efficacy studies and scientific discovery as the CHIM would recruit relevant populations with prior mycobacterial experience. As with other CHIM studies, a TB-CHIM would use a consistent infection in well characterized subjects that could be quantitatively monitored over time, dramatically reducing the number of experimental subjects needed in any study as well as allowing much shorter trials.

We reviewed the existing published TB-CHIM options by systematic review and conducted two workshop discussions—one in Europe and one in Africa—to determine if and how a TB-CHIM in Malawi could be developed. We have included a reflexivity statement ^21^ describing how we have promoted equity in our research partnership (see *Extended data* ^22^). The review and workshop discussions are reported here.

## Systematic review of human challenge studies using *M. tuberculosis*

### Methods

All attendees at the workshops have been included as authors (including as part of the collaborative group) of the manuscript; all have reviewed the manuscript and given explicit consent for their inclusion. Our PROSPERO registered ( CRD42022302785; 21 January 2022) systematic review is reported using the PRISMA 2020 checklist ^23^ (see *Extended data* ^22^) and synthesis without meta-analysis (SwIM) reporting guidelines ^24^.

### Eligibility criteria

**Inclusion criteria**: adult humans ≥18 years of age; administration of live *Mycobacterium tuberculosis* with either wild-type, or genetically modified organism (GMO); Bacillus Calmette-Guerin (BCG) interventional trials or prospective cohort studies with immune and/or microbiological end points.

**Exclusion criteria**: animal studies; publications with no primary data; interventional studies with no administration of live bacillus ( *e.g.*, viral vector vaccination trials or purified protein derivative challenge studies); and case-series, case reports and retrospective cohort studies. The studies were grouped ^24^ for narrative synthesis according to the study methodology (randomised controlled trial (RCT) *versus* non-randomised designs); challenge agent (GMO organism and BCG); administration route (intradermal, lung and oral); reporting of adverse and serious adverse events; confirmation of infection (classical culture, molecular diagnostic, and no confirmation); and immune response measurement (diverse methodologies reported so narrative synthesis applied).

### Information sources

**Figure 1.**
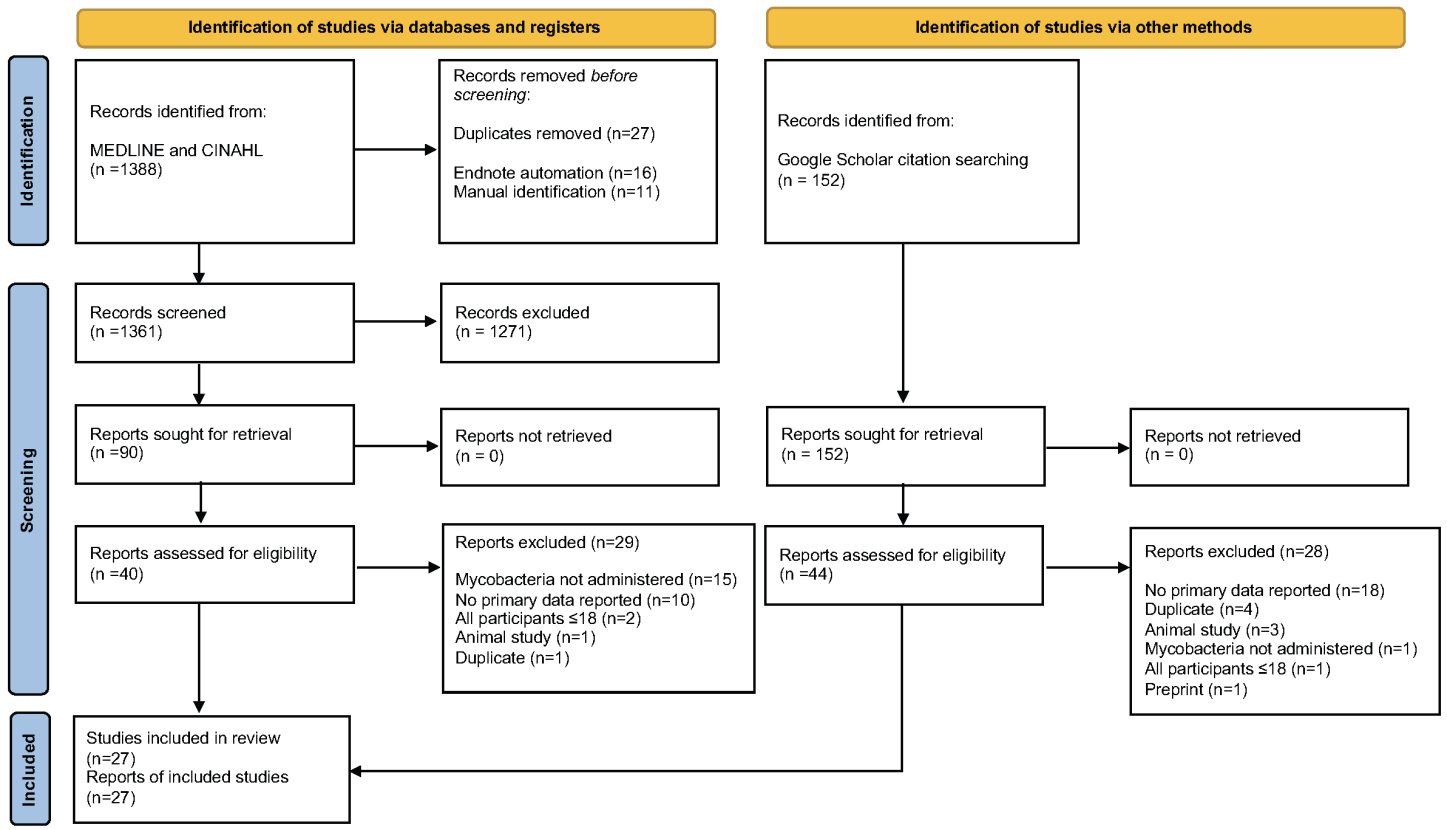
Flow diagram for inclusion of studies within the systematic review.

MEDLINE (RRID:SCR_002185) (1946 to current) and EBSCO CINAHL (RRID:SCR_022707) (1984 to current) databases were systematically searched. Table 2 describes the search terms and the search strategy. Following application of inclusion and exclusion criteria and removal of duplicates, we used Google Scholar (RRID:SCR_008878) to search citations in selected manuscripts (see CONSORT diagram, Figure 1). The final search was conducted on 3 ^rd ^February 2022.

**Table 2.** Systematic search strategy for MEDLINE. Searches 1 AND 2 AND 3 were combined for the final output. Search terms revised for matched CINHAL subject headings before searching this database.

Search	Terms
1	“mycobacterium tuberculosis” OR TB OR tuberculos* OR tuberculous OR tubercular OR pthisis OR “pulmonary consumption” OR pleurisy OR BCG OR bacill* N3 guerin OR “calmette* vaccine” OR Tuberculosis (MeSH) OR Tuberculosis, Pulmonary (MeSH) OR Tuberculosis Vaccines (MeSH) OR Tuberculosis, Pleural (MeSH)
2	“human infection model” OR “human infection stud*” OR “CHI model*” OR “CHI trial*” OR “human challenge” OR “challenge model” OR “challenge stud* OR “experimental human infection” OR “controlled human infection” OR Human Experimentation (MeSH)
3	human* OR volunteer* OR participant* OR Humans (MeSH) OR Volunteers (MeSH) OR Healthy Volunteers (MeSH)

### Selection process

After removal of duplicates, two authors independently assessed article titles, abstracts, and full manuscripts in turn to make study selection decisions. BM assessed all article titles, abstracts, and full manuscripts with secondary independent review by AC, SS and PH. Discrepancies were resolved by a third independent reviewer (DF) where these occurred. In addition, SG read all selected papers.

### Data collection

The data extraction tool was piloted by BM, AC, SS and PH independently to ensure uniformity of data collection and to refine outcome variables. Subsequently, two authors independently reviewed each manuscript and collected data. Authors then met to finalize data collection and resolve any discrepancies. No automated tools were used. The completed raw data collection tool is included in Table S1 as *Extended data* ^22^. Collected data items included: study design; setting (country); participant characteristics (number and basic demographics); challenge agent and dose; administration route; adverse events; follow up period; infection confirmation (site and method) and immunological response measurement assays. We applied relevant risk of bias assessment (Cochrane Collaboration ^25^ for RCTs and Newcastle-Ottawa Scale ^26^ for non-randomised studies) tools for included studies after data extraction. Risk of bias assessment was conducted by two independent reviewers with discrepancies resolved as previously. Given the heterogenous nature of the studies with disparate primary and secondary outcomes, we focused predominantly on methodological aspects of controlled human infection model delivery.

### Synthesis methods

We present a narrative synthesis based on study characteristics to describe how TB-CHIM studies have previously been delivered. Meta-analysis has not been conducted due to the heterogeneity of studies, study inclusion criteria and our stated aim to focus on methodological aspects of TB-CHIM delivery.

### Results

***Study selection.*** A total of 27 studies met inclusion criteria ( Figure 1). These included 15 RCTs ^16, 27– 40^; one of which was a trial protocol ^40^; and 12 non-randomised interventional studies ^17, 41– 51^. Other articles of potentially of relevance, but without primary data, did not meet eligibility criteria ^52– 61^. Only three studies were conducted in high-burden settings as shown in Table 2 and Table 3.

**Table 3.** Study characteristics for included randomised controlled trials. *Trial protocol, results not published at time of writing. X: not reported; a: mean; b: median; ID: intradermal; PO: per oral; BCG: Bacillus Calmette-Guerin; MTBVAC; live attenuated strain Mycobacterium tuberculosis; SSI: Statens Serum Institut; AERAS-422: recombinant BCG with overexpression antigens Ag85A, Ag85B, and Rv3407 and expressing mutant perfringolysin; GMO: genetically modified organism; VPM1002: recombinant BCG expressing listeriolysin, lacking urease C gene and containing a hygromycin resistance marker; rBCG30: recombinant BCG overexpressing antigen Ag85b; TICE: US brand name for intravesical BCG; CFU: colony forming units; TB: tuberculosis.

First Author	Year	Country	n	Males	Average Age	Route	Intervention	Max Dose (CFU)	Control	Follow up (days)
**Giamarellos-** **Bourboulis ^27^**	2020	Greece	150	67	80 ^a^	ID	BCG (Bulgaria)	X (0.1ml)	Saline	365
**Tameris ^28^**	2019	South Africa	18	3	29 ^a^	ID	MTBVAC (GMO wild TB)	5 x 10 ^5^	BCG (SSI)	180
**Arts ^29^**	2018	Netherlands	30	30	X	ID	BCG (SSI)	X	BCG solvent	118
**Hoft ^30^**	2017	US	86	X	X	PO & ID	BCG (SSI & Connaught)	2 x 10 ^10^ PO 5 x 10 ^5^ ID	NA	180
**Hoft ^31^**	2016	US	24	14	29 ^a^	ID	AERAS-422 (GMO BCG)	1 x 10 ^7^	BCG (TICE)	182
**Leentjens ^32^**	2015	Netherlands	40	40	21 ^b^	ID	BCG (SSI)	X	Saline	28
**Spertini ^33^**	2015	Switzerland	36	14	27 ^a^	ID	MTBVAC (GMO wild TB)	5 x 10 ^5^	BCG (SSI)	365
**Harris ^16^**	2014	UK	49	21	23 ^b^	ID	BCG (SSI)	8 x 10 ^5^	NA	42
**Grode ^34^**	2013	Germany	80	80	33 ^a^	ID	VPM1002 (GMO BCG)	5 x 10 ^5^	BCG (SSI)	180
**Wardhana ^35^**	2011	Indonesia	34	8	65	ID	BCG (Pasteur)	X	BCG solvent	90
**Hoft ^36^**	2008	US	35	13	29	ID	rBCG30 (GMO BCG)	5 x 10 ^5^	BCG (TICE)	252
**Hoft ^37^**	2000	US	48	X	X	PO & ID	BCG (Connaught)	2 x 10 ^10^ PO 3 x 10 ^6^ ID	PBS	365
**Hoft ^38^**	1999	US	66	X	X	ID	BCG (Connaught & TICE)	3 x 10 ^6^	Saline	90
**Hoft ^39^**	1999	US	48	X	X	ID	BCG (Connaught & TICE)	3 x 10 ^6^	NA	56
TBO41 trial * ^40^	2016	UK	60	X	X	Aerosol	BCG (SSI and Bulgaria)	1 x 10 ^6^	Saline	168

**Figure 2.**
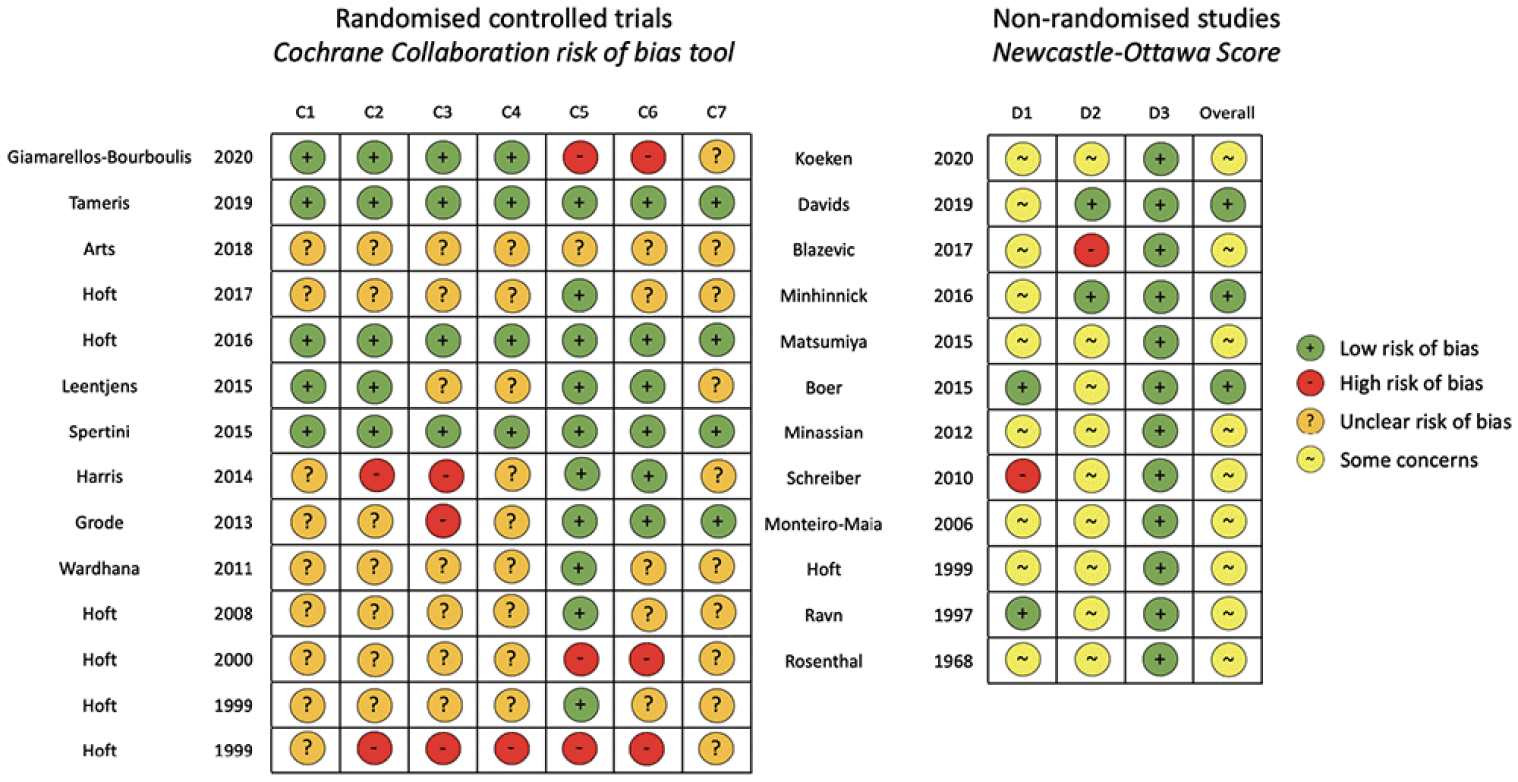
Risk of bias assessments for included studies. Cochrane collaboration risk of bias tool used for randomised trials. C1: random sequence generation (selection bias); C2: allocation concealment (selection bias); C3: blinding of participants and researchers (performance bias); C4: blinding of outcome detection (detection bias); C5: incomplete outcome data (attrition bias); C6: selective reporting (reporting bias); and C7: other bias. Newcastle-Ottawa Score used for non-randomised studies. D1: bias due to selection; D2: bias due to comparability; D3: bias due to outcome measurement; and overall score (0-3 = very high risk of bias; 4-6 = high risk of bias [some concerns]; and 7-9 = low risk of bias). Risk of bias assessment includes 26/27 studies. The excluded study is a trial protocol that has not yet reported results ( https://clinicaltrials.gov/ct2/show/NCT02709278). Matsumiya 2015 included non-randomised participants reported in Harris 2013.

***Study characteristics.*** Table 3 (RCTs, one (18 participants) from South Africa) and Table 4 (non-randomised studies, one from Brazil (6 participants) and one from South Africa (106 participants)) describe characteristics for studies selected. Primary outcome measures for the studies were diverse and as our focus was on CHIM methodology, we focused on administration route, challenge agent and dose administered in data extraction. Figure 2 describes risk of bias assessments for selected studies. Three RCTs had a low risk of bias across all assessment domains ^28, 31, 33^ and three non-interventional studies had an overall low risk of bias ^17, 42, 45^.

**Table 4.** Study characteristics for included non-randomised studies. *Matsumiya used samples taken from the same participants as Harris *et al.* ^16^. X: not reported; a: mean; b: median; ID: intradermal; Bronch: bronchoscopic installation; PO: per oral; Neb: nebulised; BCG: Bacillus Calmette-Guerin; SSI: Statens Serum Institut; TICE: US brand name for intravesical BCG; CFU: colony forming units.

First Author	Year	Country	n	Males	Average Age	Route	Intervention	Max Dose (CFU)	Follow up (days)
**Koeken ^41^**	2020	Netherlands	22	22	X	ID	BCG (Bulgaria)	X (0.1ml)	90
**Davids ^42^**	2019	South Africa	106	43	28 ^b^	Bronch	BCG (SSI)	1 x 10 ^4^	180
**Blazevic ^43^**	2017	US	5	X	X	ID	BCG (TICE)	2 x 10 ^6^	85
**Minhinnick ^17^**	2016	UK	41	13	27 ^a^	ID	BCG (SSI & TICE)	2.4 x 10 ^6^	28
*Matsumiya ^44^	2015	UK	24	X	X	ID	BCG (SSI)	8 x 10 ^5^	14
**Boer ^45^**	2015	Netherlands	12	6	24 ^b^	ID	BCG (SSI)	X	378
**Minassian ^46^**	2012	UK	40	X	X	ID	BCG (SSI)	8 x 10 ^6^	168
**Schreiber ^47^**	2010	UK	7	5	27 ^b^	PO	BCG (Moreau)	1 x 10 ^7^	365
**Monteiro-Maia ^48^**	2006	Brazil	6	1	35 ^a^	PO	BCG (Moreau)	X (100mg)	180
**Hoft ^49^**	1999	US	69	X	X	ID	BCG (Connaught & TICE)	2 x 10 ^8^	1095
**Ravn ^50^**	1997	Denmark	20	12	22 ^a^	ID	BCG (SSI)	X (0.1ml)	365
**Rosenthal ^51^**	1968	US	43	X	X	Neb	X	X	90

We focused on several aspects of CHIM study design to inform our workshops discussions. These were participant selection in mycobacterial challenge studies, challenge agent administration route and sampling post-challenge, challenge agent strains and doses for intradermal and mucosal routes, microbiological confirmation and immunological assays to confirm infection as well as reported adverse events.

***Participant selection in mycobacterial challenge studies.*** Participant inclusion criteria within the selected studies was highly variable. Two studies targeted older adults, either inpatients at discharge ^27^ or outpatients ^35^, to explore the indirect effects of intradermal BCG on subsequent development of all cause lower respiratory tract infections. Prior BCG vaccination was used very variably in different studies, either as a specific exclusion criterion ^17, 29, 31– 33, 35, 41, 43, 45, 50^; specific inclusion criterion ^28, 42^; or with an *a priori* aim to explore differences in response to mycobacterial challenge between BCG naïve and BCG exposed groups ^16, 34, 44, 46^. Several studies did not report prior BCG vaccine status ^27, 30, 36– 39, 47– 49, 51^. In addition, studies also variably used dermal response to purified protein derivative and performed interferon-gamma release assays (IGRA) as surrogates of immune response when selecting participants. As detailed in Table 3 and Table 4, most studies were conducted in geographical regions with low TB prevalence. The selection of participants for TB CHIM studies in TB endemic areas (greatest need for context-relevant therapeutics and vaccines) was more complex. In South Africa, Davids *et al.*, ^42^ categorized participants using clinical (history of treatment; presence of active disease; and household contact of index case), radiological (chest X-ray) and immunodiagnostic features (IGRA or QuantiFERON TB Gold-in tube testing) to define “protective” and “susceptible” participant phenotypes.

***Challenge agent administration route and subsequent sampling.*** Most included studies employed intradermal administration of BCG, modified BCG or modified *M.tb* and used peripheral blood derived endpoints as the most convenient route of both administration and sampling. There are a smaller number of important pulmonary studies to consider. For example, Davids *et al.*, ^42^ directly instilled BCG into a lung segment of participants with varying susceptibility phenotypes ( *e.g.*, exposed but uninfected *versus* history of recurrent TB), and compared BCG with PPD and saline instillation in alternative lung segments. That study demonstrated differential responses in bronchoalveolar lavage (BAL) immune cellular profile between the lung segments challenged and subsequently sampled. This study established the minimal BCG allowing the detection of responses and the safety of this model, the key advantage of which is interrogation of responses at the site of disease and a precise quantity of mycobacteria can be delivered to the alveolar compartment. A follow-on study to interrogate 6-month post-installation lung responses and to directly compare lung *versus* nasal *versus* skin installation is underway.

BCG administration by nebulised delivery is under investigation in a separate study ^40^ but results are not yet reported. Nebulised BCG has been tested in a historical study in both adults and children but this study was assessed at high risk of bias and the described study methodology are unlikely to meet contemporary ethical committee criteria for approval ^51^. Oral BCG administration has been explored ^30, 37, 47, 48^ but in this review these studies were assessed as at risk of bias and did not isolate mycobacteria after challenge. Oral BCG challenge, however, does show evidence of being immunoregulatory of other responses, although evidence is lacking for enhanced pulmonary defence induced by mucosally-directed vaccination ^37^.

In skin studies, the theoretical environmental risk of non-participant population exposure with intradermal administration was systematically explored in studies of both wild-type BCG ^16^ and genetically modified mycobacterial strains ^33, 34^. Persistent shedding of viable mycobacteria from ulcerated intradermal injection sites was described ^39, 43^ but this was at very low concentrations, with no recorded transmission such that this potential risk was concluded to be extremely low. Clearly skin biopsy is a valuable site for sampling in those studies, being safe and easily controlled. Reliable mycobacterial detection required large punch biopsies.

The natural route of *M.tb* infection is inhalation of aerosolized bacteria *via* the respiratory tract therefore respiratory mucosal immune responses to intradermal mycobacterial challenge may not represent natural exposure, or elicit protective pulmonary responses ^57^. Koeken *et al.*, ^41^ measured alveolar macrophage responses to intradermal BCG administration but found that induced sputum (a less invasive technique than direct bronchoalveolar lavage sampling) was associated with cellular activation but the sampling technique likely introduced artefact, impairing their ability to infer immune response to challenge.

***Challenge agent strains and doses, for intradermal and mucosal routes.*** Optimal conditions for intradermal controlled human infection with wild-type BCG have been explored, testing different strains ^17, 30, 38, 39^ and doses ^17^. The choice of BCG strain did not influence subsequent recovery of bacteria from skin punch biopsy after 14 days ^17^ nor significantly alter immune responses ^17, 30, 38, 39^ within these studies. Higher BCG doses (6 x 10 ^5^ – 2.4 x 10 ^6 ^CFU) were associated with improved bacterial detection from a day 14 skin punch biopsy with no measurable impact on adverse side effects ^17^. Early phase trials of intradermal recombinant BCG ^31, 34, 36^ and attenuated *M.tb* (MTBVAC) ^28, 33^ vaccine candidates incorporated dose escalation within their study designs.

Several recombinant BCG vaccine candidates have been compared with standard BCG. These include high dose (1 x 10 ^6^ ≥ CFU < 1 x 10 ^7^) AERAS-422 and high dose (5 x 10 ^5^ CFU) VPM1002. AERAS-422 included inserted genes to increase antigen expression and the perfringolysin gene to promote phagolysome perforation and antigen presentation—see adverse reactions section ^31^. VPM1002 included insertion of the listeriolysin gene to promote phagolysome perforation and antigen presentation and was found to be safe and immunogenic ^34^. A recombinant BCG (rBCG30) with an inserted gene to promote increased antigen expression presented at a dose of 5 x 10 ^5^ CFU was also found to be safe and immunogenic ^36^.

The attenuated *M.tb* strain MTBVAC (double deletion of independent virulence factors *phoP* and *fad*D26) was trialled at several does. The highest dose used was 5 x 10 ^5^ CFU and was found to be safe and immunogenic (n=9) when compared to standard BCG in a healthy Swiss population ^33^. Subsequently, this dose was used in nine South African adults (TB endemic area) with one serious adverse event reported (graded as unlikely related to the study vaccine) and similar reactogenicity and immunogenicity compared to BCG ^28^. The infant data from this study are not reported as these fall outside of SR inclusion criteria.

For bronchoscopically instilled BCG, a lower dose of 1 x 10 ^4^ CFU was used and found to induce significant changes to BAL cellular profiles, and antibody responses, which were not detectable below this dose. There was clear evidence of differentially expressed genes in BAL, and dysregulated proteins ^42^. Doses higher than 1 x 10 ^5^ CFU were not explored within this study. Aerosolized BCG doses up to 1 x 10 ^6^ CFU will be explored in a current (at time of writing) trial, however the results are not yet reported ^40^. Orally administered BCG has been given in much higher doses within studies reporting dosage ^30, 37, 47^, with administration of 2 x 10 ^10^ CFU in two studies ^30, 37^ and 1 x 10 ^7^ CFU in a third ^47^. The potential for differential impact of stomach acid neutralization was not systematically explored within the included studies.

***Microbiological confirmation after challenge administration.*** Microbiological confirmation of infection and clearance are critical in a CHIM. Microbiological recovery methods including sampling and processing techniques were evaluated in multiple studies within this review ^16, 17, 33, 39, 42– 44, 46^. Culture methods were the most widely used, supported by molecular techniques.

Hoft *et al.*, ^39^ used serial skin punch biopsies at days 2, 7 and 14 after intradermal BCG administration with paired histopathological evaluation to examine granuloma formation. Mycobacteria were recovered from inoculation site culture in some samples but were more reliably detected by PCR (optimal time point at D14 with detection in 6/7 samples). Subsequently, a single punch biopsy at D14 has been used by the McShane group in recent studies ^16, 17, 44, 46^. Optimisation of the punch biopsy technique is described by Minhinnick *et al.*, ^17^ who demonstrated that BCG could be recovered in all specimens (40/40) by both culture on solid agar and qPCR with strong correlation between the techniques (r=0.664) and between BCG challenge dose and subsequent biopsy CFU count (r=0.749). Harris *et al.*, demonstrated that qPCR estimated copy numbers were 1-2 logs higher (detected 48/48 samples) than cultures (detected 45/48 samples) from punch biopsy ^16^. In addition to D14 punch biopsy, Minassian *et al.*, ^46^ induced suction blisters of the BCG administration site but these were examined for immunological cellular infiltrates and microbiological results were not reported. This study demonstrated that BCG was detected in 28/28 punch biopsy specimens by qPCR and 19/28 by culture and that quantification by qPCR was a mean 1 log higher than by culture. Longitudinal swabbing of the intradermal injection site was systematically evaluated by Blazevic *et al.*, ^43^ who demonstrated that paired classical and qPCR microbiological quantification techniques demonstrated significant kinetic association. Another study, however, was not able to culture BCG by longitudinal swabbing of the injection site ^16^. Opportunistic swabbing from ulcerated/discharging BCG intradermal injection sites was applied in an early phase trial ^33^ with culture recovery (5/10 samples) for both interventional (MTBVAC, n=1) and control (BCG, n=4) mycobacteria.

In a bronchoscopic BCG installation study, Davids *et al.*, ^42^ were able to recover BCG in 6/54 (11%) of BAL microbiological samples.

***Measurement of immune response to mycobacterial challenge.*** Early BCG studies were used to describe the now well recognised induction of interferon gamma responses by mycobacteria ^38^. There are still no validated immunological correlates of protection against *M.tb* infection ^57^ and indeed most correlates fail in some subjects, particularly if dependent on a cellular response ^49^. Exciting recent progress, however, has shown the potential of transcriptomics ^62^ in defining a signature for active infection, and particularly single cell sequencing in defining immunological phenotype and linking it to clinical function ^63^. In the meantime, therefore, microbiological recovery and quantification is critically important as the primary endpoint of a TB-CHIM model.

In the studies reviewed, many different research approaches were taken to evaluate cellular and humoral responses to BCG. These include and are not limited to innate, humoral, and cellular responses but the focus has been on interferon gamma signatures at cytokine or cellular levels. The literature from non-human primate infection with *M.tb* and human studies of active TB are extensive and provide a robust background literature against which to check any human challenge findings. This will assist in validating any future TB-CHIM but are beyond the scope of this review.

### Adverse event reporting

There were no standardised reporting criteria for adverse events after mycobacterial administration in these studies, but rather an adherence to the internationally accepted adverse event, severe adverse event, and adverse events of special interest (AE/SAE/AESI) definitions in use in clinical trials. Intradermal injection of BCG was associated with local, and expected side effects following routine vaccination ^64^. The maximum follow up period for AEs was 90 days. These AEs were reported in all included studies incorporating intradermal injection and no SAEs were reported in any of the identified studies. Hoft *et al.*, ^38^ reported that all BCG intradermal injection sites were healed by 90 days, which may therefore represent a pragmatic cut off point for reporting serious adverse drug reactions (SADR) in TB-CHIM studies. Davids *et al.*, identified the minimal (safest) immunogenic dose (1 x 10 ^4^ CFU) of bronchoscopically instilled BCG ^42^. The investigators found that AEs developed in 9.4% of participants (10/106), not usually associated with bronchoscopy, and with no significant difference between BCG, purified protein derivative (PPD) and normal saline. All adverse events were reported as mild and managed in an outpatient setting.

Serious adverse events were reported, however, in studies investigating candidate TB vaccines (MTBVAC ^28^ and AERAS-422 ^31^). During a phase two trial in South Africa one participant was diagnosed with newly acquired HIV infection and aseptic meningitis three months after randomised allocation to MTBVAC vaccination ^28^. The participant was treated with empirical broad-spectrum antibiotics and anti-tuberculous therapy and was discharged from hospital five days after admission with no neurological sequelae. Six months of anti-tuberculous therapy were completed before commencement of anti-retroviral therapy with the participant reported as well with no detectable viral load at study completion by investigators. High dose AERAS-422 (n=8) was associated with varicella zoster virus reactivation in two participants ^31^ resulting in discontinuation of the vaccine development programme. Investigators within this study identified immunological and transcriptomic correlations between TB immunity and varicella zoster virus infection but no definitive causative mechanism was identified.

### Summary of systematic review findings

There are no data on controlled human infection studies using wild-type *M.tb.* There are considerable data on BCG human challenge, modified BCG and modified live *M.tb* vaccination both in carefully observed experimental studies and in vaccine trials. These data suggest that:

- BCG is a safe and acceptable experimental model of infection, using skin, pulmonary and oral routes.
- The intradermal BCG model shows some immune responses that resemble those found in TB, but intradermal BCG can only be a model of infection and not pulmonary TB disease.
- Intrapulmonary BCG may be a more promising model in that the immune responses in lungs are more representative of TB, but recovery of inoculated mycobacteria in a quantifiable manner is problematic, making this model unlikely to be useful in vaccine testing if microbiological endpoints are required. However, an alternative readout could be a host biomarker-based one including post vaccinated responses associated with protection ( *e.g.*, alveolar polyfunctional T cells, lung resident T cells *etc.*). Another approach using this model could be to use alveolar lavage cells and blood post pulmonary vaccination in an *in vitro* killing model with live mycobacteria (serving as a proxy for vaccine efficacy). Thus, different vaccines given *via* the intrapulmonary route could be compared. An additional potential here is the ability to study the route of infection (lung *versus* skin [intradermal] *versus* gut).
- Orally administered BCG is not likely to be a useful model of TB due to the high doses used, and the inability to recover mycobacteria.
- Vaccination with BCG gives variable results depending on age, global location and immune status suggesting that any CHIM data obtained from BCG human challenge must be interpreted with close attention to these parameters.
- Given the safety record of BCG, and recognising the BCG limitations, modified BCG including additional antigens or detection systems could be developed to further refine the BCG model.
- In the event that this BCG work was successful, particularly in detecting and controlling the inoculum strain, then further consideration of modified wild-type *M.tb* in CHIM models would be warranted.

## Workshop discussion of practical considerations for development of TB-CHIM

We conducted two workshops (all attendees are listed as authors or as part of the “TB Controlled Human Infection Model Development Group” in the acknowledgements section) to review the current data from human CHIM for TB, and the practical steps needed to take the work forwards. One workshop (residential, 2 days) was held in the UK (24–25 September 2019, Inglewood Manor, Cheshire, UK) and the other in Malawi (21 ^st^ June 2022, Malawi Liverpool Wellcome Programme, Blantyre, Malawi; meeting on-line and face to face). Both workshops were supportive of TB-CHIM as a worthy research aim, for the urgent reasons introduced earlier in this paper, provided that international standards of safety and volunteer consent could be achieved. Several specific topics were discussed in detail as below.

### Acceptability, specifically in UK and Malawi

In terms of practical steps, both community and stakeholder acceptability of a TB CHIM to accelerate vaccine development in any specific location (we particularly discussed the UK and Malawi) must first be assessed. There are published experiences of this CHIM acceptability enquiry process in both Kenya for malaria, and Malawi for pneumococcal carriage ^65^. The Malawi stakeholder community has previously included and would still include current CHIM researchers; the District Health Office; members of the Research Ethics Committee; potential volunteers; health experts; physicians; and public health opinion leaders ^66^.

Neither the malaria nor pneumococcal CHIM were first-in-human experiments in Africa. These models were both successfully and safely transferred from the UK after considerable experience in a non-endemic population ^67, 68^. In our Malawi workshop, there were two strongly articulated views. One was that the moral imperative to develop vaccines urgently for and in Africa made it important to pioneer these techniques in Africa, in relevant populations with endemic disease. The differences in immune experience of endemic populations might offer either increased risk or protection from the model but in either case an experiment conducted in a non-endemic region would not be informative about that risk. The alternative view was that regulators in Africa would continue to expect research protocols to be tested in research-rich environments supported by sophisticated health care systems before allowing studies of this type in settings with more limited health care systems, particularly Malawi. For example, in Kenya, guidance states that “CHIM models should be developed in maximally resourced settings before introduction to Kenya”. In any context, therefore, careful stakeholder engagement and community enquiry will be needed to determine where the community opinion lies on this spectrum. Guidance by WHO states that “there has been increased recognition of the potential value of supporting the development of infrastructure and research capacity to enable CHIS to be conducted in disease-relevant local populations in LMIC, where this meets local disease/research priorities and where such resources may not already exist.” ^69^

### Feasibility evaluation and volunteer recruitment

TB-CHIM using BCG would require appropriate clinical facilities; approved protocols; and recruitment strategy ^68^. Given that BCG is used to vaccinate the public in Malawi, offering no transmission risk, there is no requirement for containment facilities for preparation of the inoculum or to accommodate potential volunteers. Modification of BCG, or use of modified *M.tb* in future would require a detailed assessment of transmission risk and re-evaluation, as well as government regulator’s ^42^ approval for release of a genetically modified organism.

Selection of volunteers in a TB endemic area allows clinical and immunological stratification to high, middle, and low risk of past or current TB. In an African study, the risk of adverse events was highest in volunteers with positive IGRA tests (Quantiferon) and/or abnormal chest radiographs ^42^. The workshops therefore concluded that BCG CHIM in Africa should begin with IGRA negative healthy volunteers.

### Informed consent quality

Informed consent to human challenge studies in any setting is a little different to other clinical research studies because of the inoculum step proceeding towards disease, rather than a study focusing entirely on prevention, observation, or treatment. As such, information for volunteers must be clear and comprehensible, with risks explained in an appropriate manner. The quality of consent can best be obtained by evaluation of volunteer comprehension both before and after participation, with lessons learned being iteratively applied to improve the quality of the consent process in future ^70^. Further, research veterans can offer new volunteers a clear explanation of their experience in a more interesting and engaging manner than researchers, albeit with the risk of bias if only “research champions” are selected. All these methods are applicable and in use in Africa.

### Challenge agent and route of delivery

There was unanimous assent in our workshop discussions that wild-type *M. tb* could not currently be used in a pulmonary CHIM experiment because complete cure could not be guaranteed, and tissue damage remains a possibility in pulmonary models. Further, evaluation of the bacterial load of infection in the lung is not yet possible ^42^. The development of a TB-CHIM must therefore consider other agents, primarily BCG, modified BCG and modified *M.tb.*

A BCG model is the currently preferred design from which to start a mycobacterial CHIM. BCG has been administered to humans for many years by intradermal, oral, and pulmonary delivery. Intradermal and oral delivery are well trusted as safe, having been used to vaccinate billions of people world-wide ^71^. Intradermal BCG has been carefully studied in clinical trials as the preferred control in vaccine studies of novel anti-tuberculous vaccines that are also injected ^28, 31, 33, 34, 36^. Further, the optimal dose for immunological discovery experiments has been defined and optimised for intradermal studies ^17, 43, 46^. Reservations remain about this model remain, however, as protection from cutaneous infection may not represent pulmonary protection, and the punch biopsies of the skin are too invasive for repeated serial sampling. There are good data, however, that show the molecular signature of skin biopsies from tuberculin skin test (TST) sites and lungs closely reflect each other both in healthy adults and subjects living with HIV ^72^. Exaggerated IL-17 and Th17 responses found in patients with TB compared to subjects with subclinical disease drive the pulmonary damage and are also found in TST but not normal skin ^73^. Further, dose of BCG delivered, inflammatory response and inhibition of BCG growth *in vivo* ^74^ have all been shown to be closely related, suggesting a useful model ^44^. Although successful testing of a vaccine in development has not yet occurred with this model, a vaccine evaluation of MVA85A showed no vaccine effect in the BCG CHIM, consistent with the clinical trial of the same vaccine ^16^.

The oral BCG route requires very high dosing ^30^, is non-physiological and minimally immunogenic of the systemic compartment, albeit strongly inducing of mucosal immunoglobulin ^30^. The pulmonary bronchoscopy instillation route is technically difficult and particularly challenging to determine post-inoculation infection ^30^. Currently an Oxford group are working to develop an inhaled model in volunteers with previous BCG experience using post-exposure bronchoscopy to detect persistent pulmonary BCG ^75^. The instilled and nebulised pulmonary models are highly sophisticated and simpler models would be preferred in vaccine testing. A key aspect to address though, is measurement of mycobacterial killing *versus* translocation into lymph nodes.

There are modified BCG models that have shown promise in pre-clinical studies and may soon be applicable, subject to regulatory approval. A very useful potential immediate modification is to use fluorophore insertion to replace microbiological BCG measurement with fluorescent signal. This would have the enormous advantage of continuously measurable readout over time, potentially tracking mycobacterial translocation within the lymphatic system. This method, however, will require regulatory approval of the genetically modified organism; the signal detection technology; and pilot studies to show that the bacteriology and optical readouts correlate tightly within a measurable range.

Conditionally replicating or suicide strains of *M.tb* are also in development, needing some further testing in animal models to be sure of safety and stability of the strains, along with assured suicide in human subjects on removal of the conditioning agent. It is highly probable that such strains, together with appropriate short-course treatment would be very safe. Although there is a theoretical risk of long-term mycobacterial persistence in pulmonary and extrapulmonary sites ^76^, this risk is low, and steadily decreases each year after exposure. If mycobacterial detection challenges could be overcome using the BCG model, this would mitigate some of the safety challenges with a future conditionally replicating *M.tb* model.

### Different CHIM models that could be applied to TB

The pathogenesis of pulmonary TB leads from primary infection to pulmonary granuloma formation, which may lead to extensive caseation, cavitation and transmission at one extreme, whilst at the other extreme, successful walling off of the infection and eventual sterilisation. In between, pathogenesis may lead to walling off, but failure of sterilisation leading to the risk of subsequent recurrence of disease ^7, 77^. Re-infection with a new strain remains possible, but disease is often immunising and re-infection in otherwise healthy adults is considered less common. Many cured patients following TB, however, have longstanding residual lung damage demonstrable by both CT scanning and pulmonary function testing. TB-CHIM models of pulmonary disease might similarly result in lung damage and would therefore be unethical. TB-CHIM of primary infection, or re-infection followed by full early natural sterilisation should be considered. TB-CHIM of primary infection would have limited applicability to TB-endemic regions where most people develop measurable anti-mycobacterial immune responses at a very young age. A TB-CHIM of value in Africa would therefore be a model of re-infection, either resulting in natural sterilising immunity in otherwise healthy control subjects or the CHIM being controlled at an early stage to prevent any tissue damage. At this point, intradermal models are accessible and pulmonary models are less so. The main purpose of the CHIM would be to evaluate vaccines as a means of preventing re-infection.

Non-human primate models of virulent *M.tb* disease are critical in vaccine research and pathogenesis discovery ^78– 80^. The macaque is susceptible to severe TB and intravenous BCG has been shown to be protective, with protection associated with greatly increased numbers of antigen specific CD4 T cells in the lung and bronchoalveolar lavage ^81^. Repeated limiting (low) dose (RLD) infection models ^82^ have increased the sensitivity of this model with less severe disease that has allowed comparison of protective effects from less effective vaccines ^83^. The RLD approach may be of great benefit in developing safe human challenge models, and the responses observed in NHP will inform future TB-CHIM design and aims.

### Mycobacterial confirmation

Mycobacterial confirmation of BCG challenge has been consistently possible in some studies using homogenised biopsy tissue cultured on Middlebrook agar for 5 weeks ^17^. Accurate determination of copy number has been determined using PCR techniques developed in Oxford and applied to the same biopsy tissue. BCG detection on swabs of purulent indurated BCG inoculation sites has been possible but not in a quantitative manner suitable for vaccine evaluation ^43^. Culture and PCR from nasal fluid and BAL, correlating with immune responses in these compartments has been possible, but BCG and immune response detection in stools are problematic ^37^. Overall, given the preference for intradermal models, optimisation of less invasive or micro-biopsy sampling and culture/PCR is the immediate priority, with transfer to an optical readout being a most appealing future prospect.

### Immunogenicity endpoints

It is very difficult to make an early diagnosis of pulmonary *M.tb* infection because the mycobacteria are not only contained in macrophages, but also surrounded by a ball of inflammatory cells and fibroblasts ^7, 77^, long before the formation of sterilising granulomas, which themselves show variable structure and function ^84^. Very recent breakthrough studies of exhaled aerosol have demonstrated whole mycobacteria in patients with subclinical disease ^85^ but this is a recent discovery; identification is by morphology rather than culture; the application is not yet widespread. In the absence of microbiological endpoints, vaccine efficacy studies have utilised the conversion of previously interferon-gamma-release-assay (IGRA) negative recruits to become IGRA positive as confirmation of infection ^10^. This endpoint has limitations as the immunological response to *M.tb* shows variation between subjects, and can be altered by concurrent infection ( *e.g.*, HIV and COVID-19). Nevertheless, just as in prevention of infection vaccine trials, an interferon-based signal is currently the most reliable endpoint in a TB-CHIM, whether it be a release assay or a transcriptome-based assay. Alternative endpoints may potentially include measurement of polyfunctional T cells in the blood and resident in the lung. Transcriptomic studies have shown potentially improved precision in predicting the progression of subclinical (“latent”) disease to active disease ^62^, and these studies have subsequently been confirmed with PET/CT scanning to confirm current active infection in subjects lacking any symptoms ^86^.

In the development of a TB-CHIM, consideration must be given to compartmental differences in immune response. Circulating blood lymphocytes, respiratory tract mucosal tissue, bronchoalveolar lavage ^87^ and skin will exhibit different populations and percentages of macrophage, T cell and innate cell phenotypes, albeit with substantial overlap. Any endpoint must be validated for reproducibility and reliability in reporting infection, ideally by comparison with a microbiological endpoint.

### Vaccine testing and power calculation

Many novel vaccine development strategies are in advanced discovery stages, even reaching pre-clinical and phase 1, first in human evaluation. These include repeat BCG, modified BCG including *M.tb* antigens ^88^, modified *M.tb*, vector delivered antigens and RNA vaccines, delivered by a variety of routes, with and without BCG boosting ^6^. Pre-clinical evaluation can include transcriptional profiling *ex vivo*, or in animal models, computer modelling and human studies. If an intradermal BCG was used as a vaccine testing TB-CHIM, what would be the power of this study? In a proof-of-concept design, Harris *et al.*, used 12 subjects in each of four groups to compare BCG naïve/vaccinated and MVA85A vaccine *vs.* placebo ^16^. No power calculation was included in this study, and although a study using area under curve of microbiological endpoints would be ideal, multiple biopsy and multiple bronchoscopy endpoints are not likely to be feasible in a TB-CHIM. To be most useful, TB-CHIM studies must report accurate, reproducible data with a much smaller number of subjects than clinical trials—for example, a maximum of 200 subjects tested.

### Antimicrobials and drug discovery

After many years of frustration, there are now more than 25 new drugs or drug combinations to treat TB in a registered clinical pipeline ^89^. It is widely accepted that combination therapy has been successful in limiting the emergence of drug-resistant TB (MDR-TB) by the targeting of distinct essential biological functions. Furthermore, with the use of combination therapies it has been possible to shorten treatment duration and reduce relapse. However, identifying optimum combinations from a pharmacokinetic and pharmacodynamic perspective is not without significant challenges, and whilst *in vitro* and *in vivo* (animal) pre-clinical models that assess anti-TB drug activity are available, the combination of models that is most predictive of clinical treatment outcome remains unclear. This forms a significant barrier to the prioritization of promising drug regimens and there remains therefore significant room to improve the evidence base prior to commitment to Phase II/III clinical studies. A human challenge model that can be used to uncover drug combination synergies that translate clinically has the potential to accelerate the TB drug development pipeline. The use of a TB-CHIM to test drugs in development has not yet been described and a BCG model has not been used in this way. Anti-tuberculous treatment is offered to patients developing the rare clinical presentation of disseminated BCG disease ^90^. It would therefore be reasonable to expect a TB-CHIM using intradermal BCG to report reduced mycobacterial growth or accelerated clearance when treated with effective regimens or potentially useful novel agents. It is also reasonable to expect that this model would be safe given the published literature reviewed above.

### Scientific opportunity

In addition to testing vaccine efficacy and drug potency against mycobacteria, there could be wider scientific interest in a cutaneous TB-CHIM. There are increasing data to show that BCG modifies the human response to infection *via* interferon dependent mechanisms ^29^, by epigenetic modifications and particularly by modification of macrophage function—“trained immunity” ^63^. Early publications showed increased survival in young children attributable to protection from respiratory infections, and more recent work has confirmed both resilience to respiratory viral infections in older adult subjects ^27, 35^, and enhanced response to influenza vaccination ^32^. TB-CHIM using a skin BCG model would potentially allow sophisticated new techniques to be applied indirectly to determine non-specific effects of BCG in defence against respiratory infection ^63^.

### A controlled human infection model of TB in Malawi - could it last to utility?

Currently, multiple controlled human infection models are being established in populations with endemic infectious disease throughout the world including *Streptococcus pneumoniae* in Malawi ^91^, falciparum malaria in Kenya and Gabon, vivax malaria in Thailand, hookworm in Brazil; schistosomiasis in Uganda, rotavirus in Zambia (using live vaccine not wild-type virus) and shigella in Kenya. Our group has successfully established the controlled human infection model for *Streptococcus pneumoniae* infection in Malawi. The process of introducing this model included robust stakeholder engagement activities ^65, 70^; assessment by both Malawi and UK national ethical and regulatory bodies ^68^; and evaluation of participant acceptability ^67^ during the conduct of our feasibility study ^91^. Informed by this work, we have now completed a trial to test the efficacy of licensed pneumococcal conjugate vaccines and measure immunological responses in the Malawian context ^92^. The team conducting this study included Malawian doctors, nurses, data scientists, microbiologists, immunologists, and social scientists. Capacity development of this team was identified as a critical determinant of the longevity of this newly established tradition of CHIM research in Malawi.

There is a longer established tradition of TB research in Malawi, including the detailed study of mucosal immunology ^93, 94^. TB remains a major cause of morbidity and mortality ^95^ with public health interventions designed to control endemic infection impaired by the COVID-19 pandemic ^96^. Introduction of a highly efficacious TB vaccine could have transformative public health benefits. There is both the clinical and laboratory research capacity and the driving need for the TB-CHIM in Malawi, if acceptability, feasibility, and practical challenges can be overcome ^65^.

## Conclusions and next steps

There is strong support for a TB-CHIM in the global research community. There is sufficient research capacity and local support to plan a path to develop a TB-CHIM for vaccine and drug testing in both the UK and Malawi. TB controlled human infection models in skin and lung have been established in both the United Kingdom, USA and South Africa using the BCG strain with robust participant safety data reported. There are, however, considerations of risk, advantage, and disadvantage in human infection models of TB infection in Africa that require local and expert consideration. Using a pathway for introduction of a relevant and de-risked controlled human infection model (cutaneous BCG) to Malawi for the first time, we now seek to explore if TB-CHIM studies would potentially be acceptable in principle. As part of our stakeholder engagement, we will co-create the optimal design for the Malawian setting, discussing the various trade-offs for each potential approach and exploring local acceptability for each model.

## Data Availability

All data underlying the results are available as part of the article and no additional source data are required. Harvard Dataverse: CHIM SR extended and underlying data. https://doi.org/10.7910/DVN/U8IIWZ ^22^. This project includes the following extended data: PRISMA_2020_checklist.docx Reflexivity statement - TB CHIM.docx Table S1.xlsx Data are available under the terms of the Creative Commons Zero "No rights reserved" data waiver (CC0 1.0 Public domain dedication).

## Author information

***TB Controlled Human Infection Model Development Group***

Thomas Baer ^1^, Peter Banda ^2, 3^, David Barr ^4^, Emma Carter ^5^, Tarsizio Chikaonda ^2^, Andrea Collins ^5^, Graham Cooke ^6^, Dingase Dula ^2^, Fred Fyles ^5^, Kristine Guinn ^7^, Willem Hanekom ^8^, Marc Y.R. Henrion ^2, 5^, Maideyi Kanjewe ^2^, Nitya Krishnan ^6^, David G. Lalloo ^5^, Fiona Marston ^5^, Andrew McCallum ^6^, Joseph Mfutso-Bengo ^3^, Alfred Muyaya ^2^, John Ndaferakhande ^2^, Brian Ngwira ^2^, Edna Nsomba ^2^, Shaun Pennington ^5^, Anil Pooran ^9^, Ryan Robinson ^5^, Eric Rubin ^7^, David Russell ^10^, Jamie Rylance ^2, 5^, Hannah Scott ^11^, Neema Toto ^2^, Britta Urban ^12^, Claire Waddington ^6^, Steve Ward ^5^, Robert J. Wilkinson ^9^, Tom Wingfield ^5^, Angela Hyder-Wright ^5^.

1. Stanford Photonics Research Center, California, USA

2. Malawi-Liverpool Wellcome Trust Clinical Research Programme, Blantyre, Malawi

3. Kamuzu University of Health Sciences, Blantyre, Malawi

4. University of Glasgow, Glasgow, Scotland

5. Liverpool School of Tropical Medicine, Liverpool, UK

6. Imperial College, London, UK

7. Harvard T.H. Chan School of Public Health, Harvard University, Boston, USA

8. Africa Health Research Institute, KwaZulu-Natal, South Africa

9. University of Cape Town, Cape Town, South Africa

10. Cornell University College of Veterinary Medicine, New York, USA

11. The Jenner Institute, University of Oxford, Oxford, UK

12. Oxford Vaccine Group, University of Oxford, Oxford, UK
