# Supplementary material for: Practical considerations for a TB controlled human infection model (TB-CHIM); the case for TB-CHIM in Africa, a systematic review of the literature and report of 2 workshop discussions in UK and Malawi
Source: Wellcome Open Res. 2023 Jun 12;8:71. Originally published 2023 Feb 10. [Version 2] doi: 10.12688/wellcomeopenres.18767.2 (PMC10064019; doi:10.12688/wellcomeopenres.18767.2)
Supplement: Peer review history

# Reviewer report: Reviewer response for version 1

**DOI:** 10.21956/wellcomeopenres.20808.r54859

**Published:** 2023-03-30

**Authors:** Meta Roestenberg

**Affiliations:** 1 Malaria Research Group, Department of Parasitology, Leiden University Medical Center, Leiden, The Netherlands

The article presents a thorough literature review and report of several discussions between the authors on the next steps regarding a TB CHIM model. Given the impact of TB and the difficulty of progressing novel drugs and vaccines along the clinical development pipeline, the question which is being addressed is extremely timely and important. Although I feel the review was done robustly, and the results are carefully presented, as well as the discussion, the article could benefit from some additional structuring to make the arguments clear for an unfamiliar reader. Already in the introduction the authors state the challenges which come with establishing TB-CHIM (which are substantial):

- Safety aspects related to eradication, treatment, serious adverse evens, recurrent infection.
- Validity of the model: e.g. are modified strains such as BCG representative of TB infection?
- Endpoint measures: how to reliably detect infection (and preferably quantify it over time).

Although these aspects are somewhat discussed, the main focus of the review is on route of administration, participant selection, doses and immune responses. It would have been helpful to first address the proposed challenges and provide clear insights into the ultimate compromise of a human model for TB and describe how routes of administration are relevant in the context of these questions. 

 In the "conclusion and next steps" section, the author seem to settle on cutaneous BCG as the preferred model to be taken forward for testing into endemic areas. However, why this was chosen exactly is less clear from the current text. P.16 states: "a BCG model is the currently preferred design…", but p.15 also has some limitations “intradermal BCG can only be a model of infection, not pulmonary TB disease. Although I fully appreciate (and support) the choices the authors are making, I think it would benefit readers with unfamiliar background if the pros and cons were clearly stated and summarized in the concluding paragraph.

 Some additional (minor) comments:

- The summary of immunological findings are only relevant as endpoint to a CHIM if these findings are somehow a correlate of protection. If this is not the case, then this is interesting from a scientific perspective but does not necessarily accelerate the vaccine/drug development. This should then be stated.
- The need for a TB-CHIM in Africa is scientific (as the authors describe) but I think also has a very important strategic aspect in providing endemic sites with tools to take a leading position in the clinical development of novel products for TB. This can be added to page 9.
- Page 14 section on microbiological confirmation lacks discussion on the quantification of microbiological endpoints. This would greatly increase the power of the model to detect differences between vaccinated/treated individuals and placebo controls.
- Page 17 first paragraph addressed "TB-CHIM" in a very general way – I assume challenge with BCG (intradermal?) is the methodology which is referred to here?
- Page 17 second paragraph: the utility of NHP models is an important consideration in the relevance or necessity of a CHIM model. I thus suggest to move this section up (to the intro) then describe what the added value of a human model in addition to the existing NHP models would be.

Are the rationale for, and objectives of, the Systematic Review clearly stated?

Yes

Is the statistical analysis and its interpretation appropriate?

Not applicable

Are sufficient details of the methods and analysis provided to allow replication by others?

Yes

Are the conclusions drawn adequately supported by the results presented in the review?

Yes

Reviewer Expertise:

Controlled human infections

I confirm that I have read this submission and believe that I have an appropriate level of expertise to confirm that it is of an acceptable scientific standard.

# Response

**Published:** 2023-06-02

**Authors:** Ben Morton

**Affiliations:** Liverpool School of Tropical Medicine, Malawi

In the "conclusion and next steps" section, the author seem to settle on cutaneous BCG as the preferred model to be taken forward for testing into endemic areas. However, why this was chosen exactly is less clear from the current text. P.16 states: "a BCG model is the currently preferred design…", but p.15 also has some limitations “intradermal BCG can only be a model of infection, not pulmonary TB disease. Although I fully appreciate (and support) the choices the authors are making, I think it would benefit readers with unfamiliar background if the pros and cons were clearly stated and summarized in the concluding paragraph.  

**Thank you for this carefully considered review. We agree that further explanation would be beneficial and have now included an additional table to outline the pros and cons around the choice of design.**

 Some additional (minor) comments:

- The summary of immunological findings are only relevant as endpoint to a CHIM if these findings are somehow a correlate of protection. If this is not the case, then this is interesting from a scientific perspective but does not necessarily accelerate the vaccine/drug development. This should then be stated.

**We agree. We have now made this point clearer in the manuscript in the immunogenicity endpoints section.**

- The need for a TB-CHIM in Africa is scientific (as the authors describe) but I think also has a very important strategic aspect in providing endemic sites with tools to take a leading position in the clinical development of novel products for TB. This can be added to page 9.

**We agree completely with this sentiment and have now added a statement to this effect within the “Potential for a TB-CHIM” section.**

- Page 14 section on microbiological confirmation lacks discussion on the quantification of microbiological endpoints. This would greatly increase the power of the model to detect differences between vaccinated/treated individuals and placebo controls.

**We agree that robust quantification is required to detect differences between vaccinated/treated individuals and placebo controls. We have now highlighted the importance of this within the section.**

- Page 17 first paragraph addressed "TB-CHIM" in a very general way – I assume challenge with BCG (intradermal?) is the methodology which is referred to here?

**We have reworked this paragraph to provide more clarity**

- Page 17 second paragraph: the utility of NHP models is an important consideration in the relevance or necessity of a CHIM model. I thus suggest to move this section up (to the intro) then describe what the added value of a human model in addition to the existing NHP models would be.

**We have now moved this paragraph to the introduction section and described what the added value of the human model would be.**

# Reviewer report: Reviewer response for version 1

**DOI:** 10.21956/wellcomeopenres.20808.r55174

**Published:** 2023-03-29

**Authors:** Wiebke Nahrendorf

**Affiliations:** 1 Institute of Immunology and Infection Research, University of Edinburgh, Edinburgh, UK

This article explains the need for a controlled human infection model (CHIM) of tuberculosis (TB). It is part systematic review of clinical trials where *Mycobacterium tuberculosis *or the BCG vaccine were administered to adults (27 studies are described, no meta-analysis conducted) and part opinion of the TB-CHIM development group on the best way forward.

 No doubt safe TB-CHIM would accelerate vaccine development to alleviate morbidity and mortality caused by TB. It is great that this Malawi-UK collaborative team shares their considerations at the beginning this process. The paper contains a wealth of information but readers would find it more quickly if the structure was streamlined and repetition avoided.

 In my mind it would be:

1. Rationale for TB-CHIM;
2. Open questions and then for each question:
  i) review of literature - can the answer to the question be found?
  ii) interpretation - which aspects are unknown?
  iii) how does the committee propose to answer these open questions to move forward? This last part is a little weak at present - more detail and timelines are required.

From this structure it would then be great to include a summary Figure - key points in visually appealing way - so people can put it up in their presentations to broadcast your message!

Are the rationale for, and objectives of, the Systematic Review clearly stated?

Yes

Is the statistical analysis and its interpretation appropriate?

Not applicable

Are sufficient details of the methods and analysis provided to allow replication by others?

Yes

Are the conclusions drawn adequately supported by the results presented in the review?

Yes

Reviewer Expertise:

Controlled human malaria infection trials, immunology

I confirm that I have read this submission and believe that I have an appropriate level of expertise to confirm that it is of an acceptable scientific standard.

# Response

**Published:** 2023-06-02

**Authors:** Ben Morton

**Affiliations:** Liverpool School of Tropical Medicine, Malawi

We thank the reviewer for their positive feedback and careful review of our manuscript. We agree that the structure of the piece could potentially be presented in a more accessible way. We have considered this suggestion carefully but feel that restructuring in this way would weaken our reporting from the workshops in Liverpool and Blantyre. We feel it is important to emphasise these stakeholder discussions within the piece. These demonstrate our engagement on this important but potentially controversial area with co-creation of context-sensitive approaches to progression.

# Reviewer report: Reviewer response for version 1

**DOI:** 10.21956/wellcomeopenres.20808.r54857

**Published:** 2023-03-08

**Authors:** James McCarthy

**Affiliations:** 1 Peter Doherty Institute, The University of Melbourne, Melbourne, Victoria, Australia

The authors present a well- documented systematic review of human infection challenge studies with Mycobacteria, focussing on vaccines for tuberculosis. Their review has been undertaken according to the PRISMA guidelines, and is there is appropriate coverage of the potential role of challenge studies in vaccine selection, identification of immune correlates, possible drug efficacy studies and more fundamental questions around pathogenesis and immunity.

 The review summarises the different mycobacterial CHIM models and presents the results in a clear logical manner. The diversity of models, study endpoints and possible correlates of protection are well covered, as are volunteer safety issues. Specific technical challenges of the models, namely choice of challenge agent BCG/GMO etc, route of infection, endpoint selection (quantification of mycobacteria, immune correlates), other factors such overall feasibility of the model and transmission risk are all covered.

 The powerful argument that there is an imperative that such translational research be undertaken in disease-endemic low and middle income countries, such as Malawi, is well articulated. It was pleasing to see that the second workshop was conducted there and local stakeholders are included in this process and authorship.

 This review would be strengthened if the issue of how a TB CHIM would provide a convincing case for continued product development (or termination) beyond the challenge study. In malaria and some bacterial and viral challenge models, such systems have established themselves a go-no-go stage gates for vaccine or drug development. The authors conclude that at present the ID BCG infection route is currently the most practical methodology. One is left with the impression that this model as currently used is not entirely fit-for-purpose to down select vaccines. Given the issues the authors highlight, including variability in study design (e.g. dose, recovered mycobacteria detection methods [culture, PCR etc]) and diverse endpoints), the paper would be strengthened if it also made recommendations in regards to standardisation around these parameters, and what development work is a priority to make a model fit-for-purpose.

 Implementing the ID BCG model in a high burden setting may provide very useful collateral information, but will it provide the evidence base to advance or stop the development a vaccine along the critical path to registration? A critical summary of research priorities, perhaps in table form of ways that such models can assume a place would add value to the review. For example, do we need to wait for GMO challenge strains, new assays, new approaches to define study endpoints, or new routes of administration such as aerosol?

 Minor comments:

- The literature search was carried out more than a year ago. Have any key studies been reported since then?
- I am aware of two prospective studies in the 70s where BCG was administering via aerosol to lung cancer patients; these were not included in the results. They both appear to meet the inclusion criteria in the paper. Is this because the primary endpoint for these studies was safety/lung cancer treatment? Immune responses are reported in the studies e.g. skin test conversion.
  - Cusumano CL, Jernigan JA, Waldman RH. Aerosolized BCG (Tice strain) treatment of bronchogenic carcinoma: phase I study. *J Natl Cancer Inst. *1975;55(2):275-279 ^1^.
  - Garner FB, Meyer CA, White DS, Lipton A. Aerosol BCG treatment of carcinoma metastatic to the lung: a phase I study. *Cancer. *1975;35(4):1088-1094 ^2^.
- Table 3: there are two errors regarding the TB041 trial. As per the published protocol, the control group was not saline but rather intradermally administered BCG. Also the maximum aerosolised dose was 1x10 ^7 ^CFU not 1x10 ^6^ CFU (also stated incorrectly on page 14, first paragraph.)
- It would be good to standardise manuscript references or clinicaltrials.gov number (when unpublished) when referring to CHIM studies in Africa (p.9 second paragraph). Some assertions are made without any references i.e. page 9 third paragraph – references would be helpful here. Especially for assertions such as "…(TB) exposure increases susceptibility to mycobacterial infection”.
- The authors mention that large biopsies may be required to recover BCG in ID BCG CHIMS but clarification of what “large” means would be helpful. Do the authors believe this to be a problem. i.e. increased AEs, requirement for suturing etc? Does this affect consideration of the utility of an ID CHIM models in future.
- The authors propose that the PO administration is not useful model. A more explicit statement why would be helpful. Do the high doses used pose safety concerns? Is it because of inability to recover mycobacteria (as noted by the authors in the conclusion), or is it is not a common route of infection (ileal TB being rare).
- Duplicate use of remain page 16: “Reservations remain about this model remain,”
- The ordering of the clinical trials in tables 3 and 4 is not consistent (table 3 appears to be alphabetical, while table 4 is reverse chronological). I prefer a chronologic order.

Are the rationale for, and objectives of, the Systematic Review clearly stated?

Yes

Is the statistical analysis and its interpretation appropriate?

Yes

Are sufficient details of the methods and analysis provided to allow replication by others?

Yes

Are the conclusions drawn adequately supported by the results presented in the review?

Partly

Reviewer Expertise:

Human challenge models

I confirm that I have read this submission and believe that I have an appropriate level of expertise to confirm that it is of an acceptable scientific standard, however I have significant reservations, as outlined above.

## References

[1] : Aerosolized BCG (Tice strain) treatment of bronchogenic carcinoma: phase I study. *J Natl Cancer Inst* .1975;55(2) :275-9 169370

[2] : Aerosol BCG treatment of carcinoma metastatic to the lung: a phase I study. *Cancer* .1975;35(4) : 10.1002/1097-0142(197504)35:4<1088::aid-cncr2820350411>3.0.co;2-2 1088-94 10.1002/1097-0142(197504)35:4<1088::aid-cncr2820350411>3.0.co;2-2 234787

# Response

**Published:** 2023-06-02

**Authors:** Ben Morton

**Affiliations:** Liverpool School of Tropical Medicine, Malawi

This review would be strengthened if the issue of how a TB CHIM would provide a convincing case for continued product development (or termination) beyond the challenge study. In malaria and some bacterial and viral challenge models, such systems have established themselves a go-no-go stage gates for vaccine or drug development. The authors conclude that at present the ID BCG infection route is currently the most practical methodology. One is left with the impression that this model as currently used is not entirely fit-for-purpose to down select vaccines. Given the issues the authors highlight, including variability in study design (e.g. dose, recovered mycobacteria detection methods [culture, PCR etc]) and diverse endpoints), the paper would be strengthened if it also made recommendations in regards to standardisation around these parameters, and what development work is a priority to make a model fit-for-purpose.

**Thank you for these important points. We have now highlighted that further refinement of the ID CHIM model is required to develop for vaccine down selection in the “immunogenicity endpoint” section. We have also highlighted that standardisation of study design is a priority in the “vaccine testing and power calculation” section.**

 Implementing the ID BCG model in a high burden setting may provide very useful collateral information, but will it provide the evidence base to advance or stop the development a vaccine along the critical path to registration? A critical summary of research priorities, perhaps in table form of ways that such models can assume a place would add value to the review. For example, do we need to wait for GMO challenge strains, new assays, new approaches to define study endpoints, or new routes of administration such as aerosol?

**Thank you for raising this. We have now expanded the section “A controlled human infection model of TB in Malawi - could it last to utility?” section to explore how we would envisage introduction of the TB-CHIM model in Malawi.**

 Minor comments:

- The literature search was carried out more than a year ago. Have any key studies been reported since then?

**We have re-run our systematic search (24/04/2023) and no further key studies have been published since our original search.**

- I am aware of two prospective studies in the 70s where BCG was administering via aerosol to lung cancer patients; these were not included in the results. They both appear to meet the inclusion criteria in the paper. Is this because the primary endpoint for these studies was safety/lung cancer treatment? Immune responses are reported in the studies e.g. skin test conversion.
  - Cusumano CL, Jernigan JA, Waldman RH. Aerosolized BCG (Tice strain) treatment of bronchogenic carcinoma: phase I study. *J Natl Cancer Inst. *1975;55(2):275-279 ^1^ .
  - Garner FB, Meyer CA, White DS, Lipton A. Aerosol BCG treatment of carcinoma metastatic to the lung: a phase I study. *Cancer. *1975;35(4):1088-1094 ^2^ .

**Thank you for drawing attention to these two interesting studies in patients with lung cancer. Similarly, BCG is also given via the intravesical route for TCC bladder with multiple studies reporting this. We decided to exclude such studies in patients who are likely to have altered immune response to infection but neglected to include this criterion in the submitted manuscript. We have now corrected this in the revised manuscript.**

- Table 3: there are two errors regarding the TB041 trial. As per the published protocol, the control group was not saline but rather intradermally administered BCG. Also the maximum aerosolised dose was 1x10 ^7 ^CFU not 1x10 ^6^ CFU (also stated incorrectly on page 14, first paragraph.)

**We have now clarified use of intradermal BCG for the control group in the Table legend (the control group also receive aerosolised saline). We have now checked with Prof McShane (CI for TB041), personal communication, who confirmed the maximum dose was 1x10 ^7 ^CFUs. We have now corrected this issue in the manuscript.**

- It would be good to standardise manuscript references or clinicaltrials.gov number (when unpublished) when referring to CHIM studies in Africa (p.9 second paragraph). Some assertions are made without any references i.e. page 9 third paragraph – references would be helpful here. Especially for assertions such as "…(TB) exposure increases susceptibility to mycobacterial infection”.

**We have now added references as suggested and removed examples where references were not accessible.**

- The authors mention that large biopsies may be required to recover BCG in ID BCG CHIMS but clarification of what “large” means would be helpful. Do the authors believe this to be a problem. i.e. increased AEs, requirement for suturing etc? Does this affect consideration of the utility of an ID CHIM models in future.

**We have now clarified what large means (≥4mm as the standard diameter of a skin punch biopsy) and highlighted the inherent limitation of this. We have also highlighted that exploration of smaller (microbiopsies) in further development/refinement work may be beneficial.**

- The authors propose that the PO administration is not useful model. A more explicit statement why would be helpful. Do the high doses used pose safety concerns? Is it because of inability to recover mycobacteria (as noted by the authors in the conclusion), or is it is not a common route of infection (ileal TB being rare).

**We have now more explicitly highlighted in the review that the main reason we propose PO administration is not useful for this indication is due to the inability to recover mycobacteria.**

- Duplicate use of remain page 16: “Reservations remain about this model remain,”

**Corrected, thank you.**

- The ordering of the clinical trials in tables 3 and 4 is not consistent (table 3 appears to be alphabetical, while table 4 is reverse chronological). I prefer a chronologic order.

**We have now updated both tables to be in chronological order.**
